# Demographic predictors of bedtime procrastination in the Japanese population

**DOI:** 10.1007/s41105-023-00508-7

**Published:** 2024-01-18

**Authors:** Shion Miyagawa, Tomoya Sato, Shunta Maeda

**Affiliations:** 1 Graduate School of Education, Tohoku University, Miyagi, Japan; 2 Institute of Humanities and Social Sciences, Niigata University, Niigata, Japan

**Keywords:** Bedtime procrastination, Sleep, Sleep deprivation, Self-control

## Abstract

Bedtime procrastination (BP) is prevalent and problematic, with no previous study conducted in Japan. This study developed the Japanese version of the Bedtime Procrastination Scale (BPS), assessed its validity, and investigated the relationship between BP, demographic features, and sleep outcomes. Participants were divided into two samples (*N* = 252 and *N* = 630). Sample 1 involved a longitudinal study to confirm test–retest reliability of the BPS. Sample 2 involved a cross-sectional study to assess confirmatory factor analysis, criterion-related validity, construct validity, and determine the internal consistency of the BPS. The relationship between BP and demographic features (gender, age, and employment status) and sleep outcomes (Athens Insomnia Scale, sleep hours, sleep onset latency, and sleep efficiency) was investigated using Sample 2. The BPS showed good internal consistency (Cronbach’s α coefficients = .90–91), test–retest reliability (ICC = .86), and one factor model was valid; CFI = .95, TLI = .94, RMSEA = .10, and SRMR = .04. The BPS had a moderate positive association with general procrastination, moderate negative associations with self-control, sleep quality, and sleep duration on weekdays, and those who answered “yes” to the item “Do you have trouble with bedtime procrastination?” had higher BPS scores. BPS scores were moderately higher for younger participants (≤ 40 years), slightly higher for females, and non-significantly different between employment statuses. The BPS showed a significant positive correlation with insomnia symptoms, weak positive association with sleep efficiency on weekdays and holidays, and no significant association with sleep onset latencies on holidays and weekdays. This study provides new data on demographic predictors of BP in Japan. No clear effects of gender and employment status were found, but age was a strong predictor of BP, where younger age groups had a higher BP risk.

**Supplementary Information:**

The online version contains supplementary material available at 10.1007/s41105-023-00508-7.

## Introduction

Sleep deprivation is associated with lifestyle-related diseases, such as obesity and diabetes [1], psychiatric symptoms, such as depression and worry [2], and additional problems, including poor occupational performance, increased absenteeism, higher medical costs, and deteriorating quality of life [3]. Japanese people are known to sleep the least in the world [4], and sleep deprivation is a serious concern in Japan.

In recent years, bedtime procrastination (BP) has gained attention as a factor associated with sleep deprivation [5]. BP is defined as going to bed later than intended, without external circumstances preventing a person to do so, despite expecting to be worse off as a result [5–7]. BP decreases sleep satisfaction and duration [6–13]. Numerous studies have also shown that BP is associated with fatigue, depression, and worry in young adults and adults [12–18]. In a previous meta-analysis, a moderate positive correlation was seen between bedtime procrastination and daytime fatigue (*z* = 0.31) [13], and it has also been shown that BP leads to decreased psychological vitality and performance [19], so it may lead to either absenteeism or presenteeism.

Although many studies on BP have been conducted, none have been conducted in Japan. Adolescents in Asia, especially Japan, sleep less than other regions [20], the prevalence of bedtime procrastination in different demographic groups in Japan has yet to be presented. To address sleep deprivation caused by BP, investigation of the demographic risk factors associated with BP in Japan is necessary.

The relationship between BP and demographic features has not been clarified. While many studies show that BP is more common in women [5, 8, 21]. Dardara & Al-Makhalid [22] showed that BP was more common in men. Furthermore, it was suggested that BP was more predominant in younger than in older people. However, some surveys have included only college students [7, 21], with few large-scale surveys covering a wide age range. Furthermore, previous study has found no difference between students and working people [7, 9]; it seems warranted to verify whether a similar association would be noted in Japan.

In this study, we aimed to (a) validate the Japanese version of the Bedtime Procrastination Scale (BPS) [7] and (b) explore the relationship between BP and demographic factors in the Japanese population. We sought to clarify the relationship between BP and the characteristics of a large population, including young and older people. Additionally, we explored the relationship between BP and insomnia symptoms.

## Methods

### Participants

The survey was conducted from December 14–December 31, 2022, for Sample 1 and November 29–December 13, 2022, for Sample 2. Participants were recruited via an online crowd-sourcing platform (CrowdWorks) with 4.8 million enrollments. Participants were selected on a first-come, first-served basis. Informed consent was obtained from all participants. This study used two samples (Samples 1 and 2). The exclusion criteria of both samples were (a) a self-reported current history of a sleep disorder and (b) failing the attention check while answering the questionnaire [23]. The demographic characteristics of the two samples are shown in Table 1. Sample 1 was used for exploratory factor analysis, internal consistency, and to confirm the test–retest reliability of the BPS. Among those who responded to the initial survey (*n* = 252; Time 1), 189 participants completed a follow-up survey (Time 2) within approximately 14 days (Mean = 13.9 ± 0.5 days). No participants in Sample 1 met the exclusion criteria, and all 252 participants were included. The COSMIN checklist was used to determine the sample size for the study [24]. The COSMIN checklist establishes the number of people needed to validate health-related measures; for factor structure validity, internal consistency, and test–retest reliability, a minimum of 100 participants is desirable [24]. Therefore, the sample sizes for Samples 1 and 2 were set to at least 100 participants, considering the expected number of missing data.

**Table 1:** Breakdown of the demographic characteristics of Samples 1 and 2

Category	Sample 1 (%)		Sample 2 (%)
	Time 1	Time 2	
*Gender*			
Female	140 (55.6%)	101 (53.4%)	366 (57.2%)
Male	110 (43.7%)	88 (46.6%)	267 (41.7%)
Others	2 (0.8%)	0 (0.0%)	7 (1.1%)
*Age*			
18–20	2 (0.8%)	1 (0.5%)	97 (15.2%)
21–30	42 (16.7%)	30 (15.9%)	134 (20.9%)
31–40	95 (37.7%)	64 (33.9%)	138 (21.6%)
41–50	85 (33.7%)	70 (37.0%)	139 (21.7%)
51 or over	28 (11.1%)	24 (12.7%)	132 (20.6%)
*Employment*			
Full-time employment	118 (46.8%)	88 (46.6%)	260 (40.6%)
Unemployed	75 (29.8%)	52 (27.5%)	167 (26.1%)
Student	7 (2.8%)	6 (3.2%)	94 (14.7%)
Part-time employment	47 (18.7%)	38 (20.1%)	114 (17.8%)
Night worker	5 (2.0%)	5 (2.6%)	5 (0.8%)
*Having trouble with BP*			
Yes	–	–	275 (43.0%)
No	–	–	365 (57.0%)

For Sample 2, participants from five age groups were recruited. The age groups were 18–20 years (*n* = 97), 21–30 years (*n* = 134), 31–40 years (*n* = 138), 41–50 years (*n* = 139), and ≥ 51 years (*n* = 132). The breakdown of employment status by age group of Sample 2 can be found in Table 2. Sample 2 was used for confirmatory factor analysis, criterion-related validity, construct validity, and for determining the internal consistency of the BPS. Additionally, we explored the relationship between BP and demographic variables in Sample 2. Among those who initially participated in the Sample 2 survey, 13 participants who failed the attention check were removed, leaving 640 participants for the analysis.

**Table 2:** The breakdown of employment status by age group of Sample 2

Age	Full-time employment	Unemployed	Student	Part-time employment	Night worker
18–20	9	5	80	3	0
21–30	56	36	14	27	1
31–40	71	40	0	27	0
41–50	71	41	0	26	1
51–	53	45	0	31	3

### Questionnaires

#### Bedtime procrastination

The BPS used in this study [7] is a 5-point scale (1 [never] to 5 [always]) with nine items. The BPS was translated from the original English into Japanese after obtaining permission from the developer. Two native Japanese-speaking researchers in clinical psychology independently translated the original English version into Japanese. Subsequently, the translated version was back-translated from Japanese to English by an additional independent bilingual translator. No marked differences were observed between the back-translated and original versions. Finally, the BPS developer assessed the original and back-translated versions for uniformity, and recommended revisions were made. Finally, the developer rechecked the revised version and confirmed the absence of semantic differences between the original and back-translated versions. The final version of the scale can be found in the Supplementary Material (Online Resource 1).

In addition to the BPS, an anchor item was utilized to examine the test–retest reliability of the Japanese version of the BPS. AtTime 2, participants in Sample 1 responded to the question, “Has your frequency of BP increased in the past two weeks since the last survey?” on an 11-point scale (-5: decreased considerably; 0: unchanged; 5: increased considerably). To test the criterion-related validity of the BPS, scores between the group that answered “yes” and the group that answered “no” to the item “Do you have trouble with bedtime procrastination?” were compared.

#### General procrastination

The pure procrastination scale [25] was used to confirm the validity of the BPS. We used the Japanese version of this scale [26]; a 5-point scale ranging from 1 (not at all) to 5 (always), with 12 items to assess general procrastination. Cronbach’s α and ω coefficients were 0.94 and 0.95, respectively.

#### Self-control

To confirm the validity of the BPS, we used the short version of the Self-Control Scale [27]. We used the Japanese version of the Self-Control Scale [28]; a 5-point scale ranging from 1 (not at all) to 5 (very much), with 13 items to assess self-control. Cronbach’s α and ω coefficients were 0.87 and 0.89, respectively.

#### Insomnia symptoms

The Athens Insomnia Scale [29] was used to evaluate sleep quality at least three times a week during the past month. This study used the Japanese version of this scale [30]; a 4-point scale requiring responses for eight items. Cronbach’s α and ω coefficients were 0.86 and 0.90, respectively.

#### Sleep schedules

The questionnaire includes questions on the time of sleep onset, time of awakening, latency to fall asleep, and actual sleep duration. The questionnaire items were developed from the Japanese version [31] of the Pittsburgh Sleep Quality Index [32], with eight items for weekdays and holidays (sleep before holidays). We calculated sleep efficiency using these items; dividing actual sleep time by the difference between the time of falling asleep and that of waking. Among the participants in Sample 2, 90 whose sleep efficiency was not between 0 and 1 were excluded from the analysis of sleep efficiency.

#### Procedures

Participants in Sample 1 were asked to complete the BPS and answer questions about the frequency of BP. Those in Sample 2 were asked to complete the BPS, Pure Procrastination Scale, Self-Control Scale, and Athens Insomnia Scale. Sleep outcome and schedule data were also provided. The study protocol was approved by the Research Ethics Review Committee of the Graduate School of Education of Tohoku University (21-1-049). The study protocol was preregistered with the Open Science Framework, where all study data are available [33].

### Analysis

#### Preregistered analyses

Analyses regarding the reliability and validity of the BPS were preregistered and performed accordingly. The details of the preregistered analyses and hypotheses are shown in Table 3. To assess internal consistency, we calculated the α and ω coefficient of the BPS. To assess factorial validity, we conducted an exploratory factor analysis in Sample 1 and a confirmatory factor analysis in Sample 2. Before conducting factor analyses, in addition to the preregistered analysis, we conducted the Kaiser–Meyer–Olkin (KMO) test to determine how suited the data were for factor analysis. For construct validity, we assessed the correlations between BPS and general procrastination, self-control, sleep quantity, and sleep quality. To assess test–retest reliability, we used an anchor item and ICC. To examine criterion-related validity, we compared the BPS scores between individuals that answered “yes” and those that answered “no” to the item “Do you have trouble with bedtime procrastination?” using a Student’s* t* test.

**Table 3:** Preregistered analyses and hypotheses regarding the reliability and validity of the BPS

Types of reliability and validity	Predicted results
Internal consistency	The α coefficient of the BPS is approximately .90 [5], and the Japanese version of the scale is expected to show similar high internal consistency(α≧.80)
Factorial validity	By conducting an exploratory factor analysis in Sample 1 and a confirmatory factor analysis in Sample 2 based on the results of the exploratory factor analysis, a one-factor structure of the BPS is expected [5]. In the exploratory factor analysis, the result of the parallel analysis is used as the criterion for the number of factors In confirmatory factor analysis, CFI ≥ .95, TLI ≥ .95, RMSEA ≤ .06, and SRMR ≤ .08 are used as criteria for the validity of the one-factor structure in terms of model fit
Construct validity	Consistent with Kroese et al. (2014), we expect to find a moderate positive association between the BPS and general procrastination. A moderate (> = .30) negative association is noted between the BPS and self-control, sleep quantity, and sleep quality
Test–retest reliability	Consistent with Kroese et al.’s study (2016) (r = .79), a good retest reliability (between .75 and .90) is expected
Criterion-related validity	Since the BPS includes questions about the frequency of delayed bedtime, the group with a higher predisposition to delayed bedtime had significantly higher BPS scores than the group that did not

#### Exploratory analysis

To investigate the relationship of BP with demographic variables and insomnia symptoms, the correlation coefficients were calculated in Sample 2. Additionally, BPS scores were compared between age groups, genders, and employment statuses, with analysis of variance^1^ and follow-up analysis with Tukey-HSD correction. Further, to assess the level of BPS scores that would indicate maladaptive BP; the cutoff BPS score was determined via ROC analysis using the item “Do you have trouble with bedtime procrastination?” The R 4.2.2 statistical software was used for statistical analysis of the results.

## Results

### Validation of the Japanese version of the BPS

#### Factorial validity

**Fig. 1:**
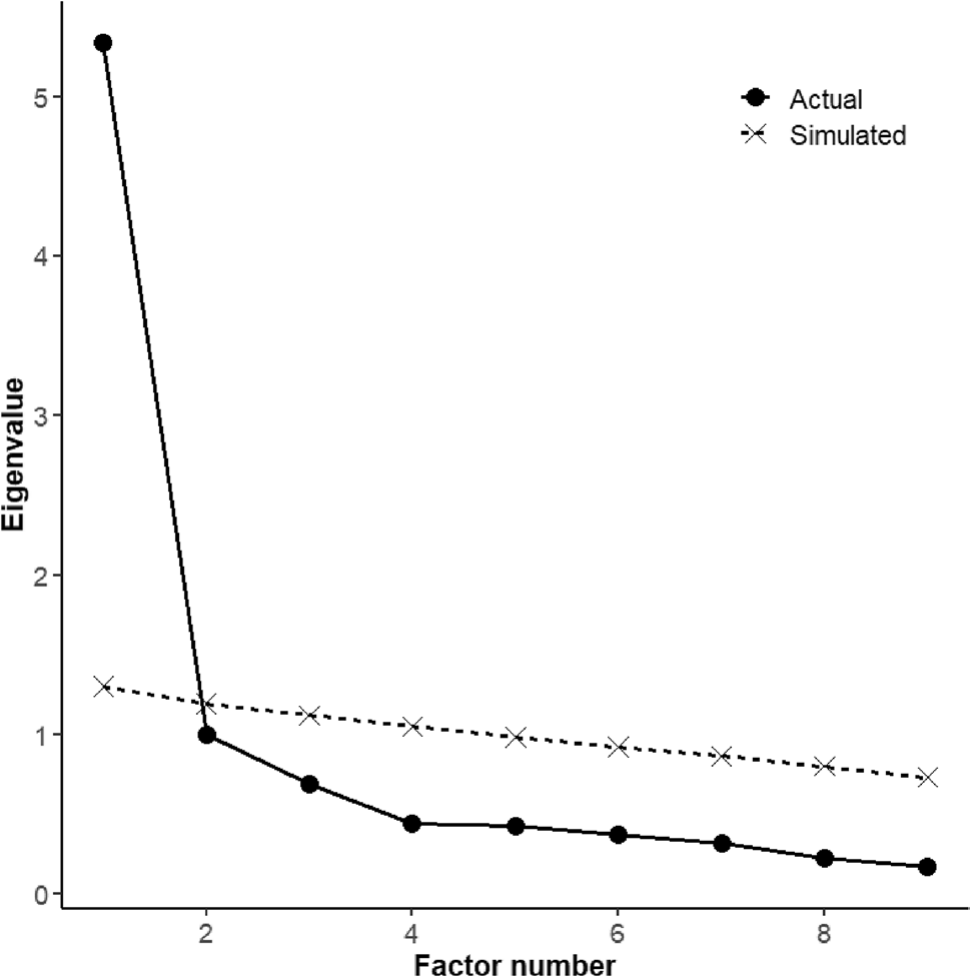
Result of parallel analysis

In Samples 1 and 2, the overall Kaiser–Meyer–Olkin MSAs for the BPS were satisfactory (MSA = .93, for both). Individual MSA indices are listed in Table 4. The results of parallel analysis using the maximum likelihood method indicated that the one-factor model was valid (Eigenvalue = 5.22; Fig. 1). The results of a confirmatory factor analysis showed CFI = .95, TLI = .94, RMSEA = .10, and SRMR = .04. Some indices did not meet the preregistered cutoffs. However, as the model showed an almost satisfactory fit, a one-factor model was accepted in this study. The factor loadings and their standard deviations are shown in Table 4.

**Table 4:** MSA indices, factor loadings, and the standard deviations for each item on the BPS

	Kaiser–Meyer–Olkin MSA		Factor loadings (SD)
	Sample 1 (Time1)	Sample 2	Sample 2
BPS 1	.92	.92	.90 (.04)
BPS 2	.45	.72	.12 (.04)^a^
BPS 3	.95	.93	.79 (.05)
BPS 4	.92	.93	.96 (.04)
BPS 5	.94	.93	.82 (.04)
BPS 6	.90	.91	.89 (.03)
BPS 7	.94	.92	.90 (.04)
BPS 8	.96	.96	.78 (.04)
BPS 9	.94	.94	.86 (.04)

^a^Although item 2 showed an insufficient MSA value, exclusion of the item did not influence the findings; therefore, the item was included in the analysis as preregistered

*BPS*  The Bedtime Procrastination Scale

#### Internal consistency

The internal consistency of the BPS was sufficient, with Cronbach’s α coefficients of .90, .91, .90 at Time 1, 2 of Sample 1, Sample 2, respectively, and ω coefficients of .92, .94, .92 at Time 1, 2 of Sample 1, Sample 2, respectively.

#### Test–retest reliability

For Time 1 and Time 2 surveys, the mean subjective change in BP was 0.44 (95% CI: 0.26 to 0.62), suggesting BP was almost stable between the two measurement points. The ICC (2, 1) for total BPS scores between Times 1 and 2 was 0.86. The minimal detectable change (MDC) 95 was 1.54. The ICC slightly increased when only participants who reported no change in BP between the measurements (*n* = 126) were included (ICC (2, 1) = .88).

#### Construct validity

Table 5 shows the means and standard deviations of the BPS and other measures, and their associations with the BPS. The BPS showed a moderate positive association with general procrastination (*r* = .47, *p* < .001) and moderate negative associations with self-control (*r* =  – .41, *p* < .001), sleep quality (*r* = .44, *p* < .001), and sleep duration on weekdays (*r* =  – .26, *p* < .001). The analysis also found a small negative association with sleep duration on weekends (*r* =  – .08, *p* = .05).

**Table 5:** Means and standard deviations of the BPS and the concurrent tests and the associations between the variables

Variable	Mean (SD)	Correlation with BP
BPS	26.71 (7.50)	–
General procrastination	28.15 (10.21)	.47*
Self-control	39.46 (9.08)	– .41*
AIS	6.52 (4.21)	.44*
Sleep onset latency (weekdays; minutes)	57.00 (137.40)	– .03
Sleep onset latency (holidays; minutes)	61.80 (142.20)	– .02
Sleep hours (weekdays)	6.44 (1.20)	– .26*
Sleep hours (holidays)	7.22 (1.40)	– .08
Sleep efficiency (weekdays)	0.89 (0.12)	– .14*
Sleep efficiency (holidays)	0.90 (0.18)	– .08*

^*^p < .05

*BPS*  The Bedtime Procrastination Scale, *BP*  Bedtime Procrastination, *AIS*  The Athens Insomnia Scale

#### Criterion-related validity

The group that answered “yes” had significantly higher BPS scores than those that answered “no” (*t* (622) =  – 19.18, *p* < .001, *d* = 1.55). The ROC analysis showed that the appropriate cutoff value was 26 points, with a sensitivity of 85.7% and a specificity of 70.7% (AUC = .864), indicating that those who scored 26 points or higher had trouble with BP.

### Exploratory analysis

#### Associations with demographic variables

**Fig. 2:**
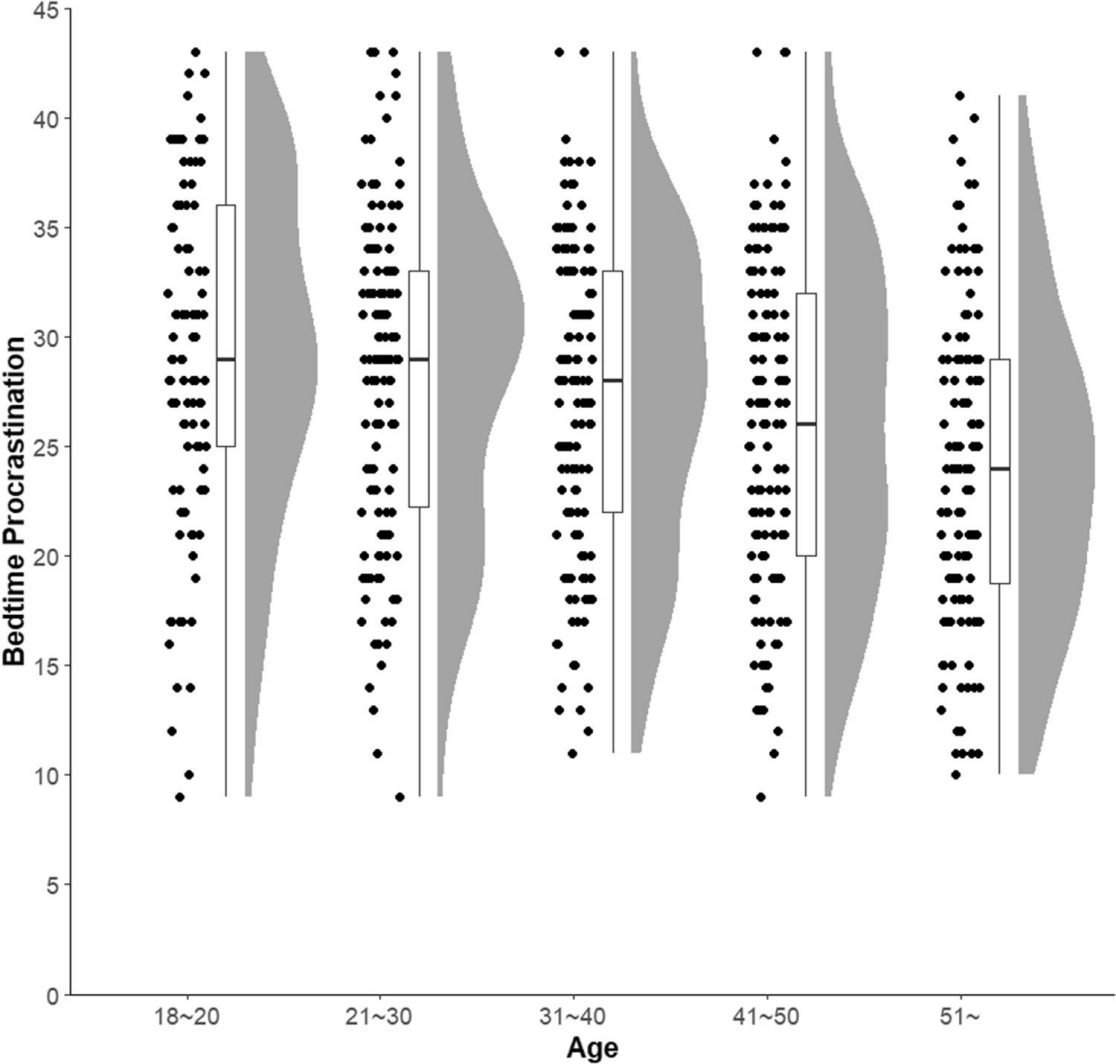
Raincloud plots for each age group on the BPS

In Sample 2, females had significantly higher BPS scores than males, with a small effect size (*t* (615) = 2.78, *p* = .006, *d* = 0.22). A significant negative correlation (*r* =  – .24, *p* < .001) was observed between the BPS and age. Analysis of variance was conducted by dividing the participants into five groups, and the results showed significant differences (*F* (4, 635) = 9.37, *p* < .001). The 18–20 years group had significantly higher BPS scores than the ≥ 51 years group (*t* (635) = 5.34, *p* < .001, *d* = 0.71) and significantly higher than the 41–50 years group, with a moderate difference (*t* (635) = 3.29, *p* = .009, *d* = 0.42). The 21–30 years group had significantly higher BPS scores than the ≥ 51 years group, with a moderate difference (*t* (635) = 4.75, *p* < .001, *d* = 0.60). Additionally, the 31–40 years group had significantly higher BPS scores than the ≥ 51 years group, with a moderate difference (*t* (635) = 3.69, *p* = .02, *d* = 0.47; Fig. 2). Regarding employment status, no significant difference was found between full-time employment, part-time employment, unemployment, and student statuses (*F* (1, 633) = 5.34, *p* = .68).

#### Association with insomnia symptoms

In Sample 2, the association between BPS and insomnia symptoms is shown in Table 5. The BPS showed a significant positive correlation (*r* = .44, *p* < .001) with the Athens Insomnia Scale. There was no significant association between the BPS and sleep onset latencies on holidays and weekdays, with a weak positive association between BP and sleep efficiency on weekdays and holidays (*r* =  – .14 and  – .08, respectively).

## Discussion

This study aimed to develop the Japanese version of the BPS and examine demographic risk factors for BP in Japan. The Japanese BPS showed good reliability and validity, confirming the preregistered hypotheses, except for some fit indices in confirmatory factor analysis. BPS scores were moderately higher for younger than older participants, consistent with a previous study [7]. Also, females showed slightly higher BP scores than males. There were no significant differences in BPS scores between students, full-time workers, part-time workers, and unemployed individuals. The BPS also showed a significant positive correlation with insomnia symptoms.

Our survey, with a wide age range of participants, confirms the role of age as a predictor of BP. Particularly, those ≤ 40 years showed higher BP levels than those > 41 years with at least moderate effect sizes, suggesting higher risk for younger individuals. The relationship between age and BP could potentially be attributed to several factors, including self-control, evening smartphone use, [19] or work-related anxiety. Among these factors, chronotype could be a predominant factor explaining the relationship between age and BP. Chronotype refers to an individual’s internal biological clock, influencing their preferred times of day for certain activities [34]. Notably, a nocturnal chronotype has shown associations with elevated BP [13] and is more prevalent in adolescents [35]. Consequently, we anticipate a decline in BP as nocturnal tendencies diminish with age.

In this study, there was no difference in BP based on employment status, consistent with those of previous studies [7]. Although it has also been reported that workload affects BP frequency [9] and full-time employees are expected to work more than others; however, other employment may have additional workloads (household chores, studies, and other engagements). Thus, the effect of their employment status may not have been apparent.

Additionally, females had higher BPS scores than males, but the effect size was small; this gender-related difference is difficult to interpret. Considering the small effect size in our study and varying findings from related studies [5, 8, 21, 22], gender might not be a strong predictor of BP; a confounder (e.g., mental health condition [36]) may be related to the gender difference.

Moreover, we found a relationship between BP and insomnia. Although BP has a possible different mechanism leading to insufficient sleep than that of insomnia [6], the relationship between BP and insomnia requires further clarification.

### Limitations

This study has several limitations. First, the small sample size influenced the investigation of certain demographic effects. Particularly, there was an insufficient number of participants who worked night shift, and the effect on BP could not be tested. A previous study revealed that night shifts tended to cause sleep deprivation [37], and examining the relationship with BP is necessary. Moreover, it would be valuable to examine the effects of different unemployed statuses (job seekers, or full-time stay-at-home parents, etc.). Furthermore, since participant selection was not separated by age and employments, investigation of the effects of each demographic, controlling for other demographic factors, was not possible. Thus, a future replication with a larger sample would be desired. Second, to reduce respondent burden, individual items were selected to assess sleep quantity on both weekdays and holidays, and the trouble with BP, however, validated measures would have been ideal. Third, we could not examine BP in very young individuals because we did not include individuals < 18 years, although the study showed that BP was most common in the youngest age group. Also, further exploration of the relationship between BP and various lifestyles is required. Finally, as we used the online survey method, unquantifiable responder biases and limited representativeness of the population may have affected the findings. It is desirable to further generalize the findings using a formal method of sampling in the future.

## Conclusion

Notwithstanding the limitations, this study provides new data regarding demographic predictors of BP in the Japanese population, which is known for short sleep duration. The absence of obvious effects of gender and employment status on BP suggests that BP might be influenced by personal lifestyle factors rather than social attributes. Nevertheless, age was a relatively strong predictor of BP in the Japanese population. Particularly, younger individuals are at a higher BP risk than those older than 40. Further research targeted at younger individuals, to elucidate the mechanism of BP, may lead to the development of an effective prevention or treatment program for BP. The Japanese version of the BPS developed in this study would be a valuable tool to quantify BP in future studies in Japan, to further strengthen the findings.

### Supplementary Information

Below is the link to the electronic supplementary material.

Supplementary file1 (PDF 319 KB)

## Footnotes

1 The “other” gender and night shift were not included in the analysis because the number of respondents was too small.
