# Supplementary material for: Demographic predictors of bedtime procrastination in the Japanese population
Source: Sleep Biol Rhythms. 2024 Jan 18;22(2):199–206. doi: 10.1007/s41105-023-00508-7 (PMC10959875; doi:10.1007/s41105-023-00508-7)
Supplement: Supplementary file 1 — Supplementary file1 (PDF 319 KB) [file 41105_2023_508_MOESM1_ESM.pdf]

Supplementary Material The Japanese version of Bedtime Procrastination Scale

次のそれぞれの質問について，あなたにあてはまる程度を 1（ほとんどない）から 5（ほとんどいつも）の間で選んでください。						
Item		ほとんどない	あまりない	ときどきある	かなりある	ほとんどいつも
1	寝床に入るつもりだった時間よりも，遅く寝床に入る。	1	2	3	4	5
2	朝早く起きなければならないとき，早く寝床に入る。	1	2	3	4	5
3	夜，消灯する時間になったら，すぐに消灯する。	1	2	3	4	5
4	寝床に入る時間なのに，まだ他のことをしていることが多い。	1	2	3	4	5
5	本当に寝床に入りたいのに，他のことで簡単に気が散る。	1	2	3	4	5
6	寝床に入るべき時間に入らない。	1	2	3	4	5
7	一定の寝床に入る時間を守っている。	1	2	3	4	5
8	寝床に入るべき時間に入りたいが，単にそうしない。	1	2	3	4	5
9	寝床に入る時間になったら，活動を簡単にやめることができる。	1	2	3	4	5

Note) Among the individual MSA indices for each item, item 2 showed an insufficient MSA value (MSA = .45 in Sample 1 at Time 1). However, as the exclusion of item 2 did not make a large difference in findings, item 2 was included in the Japanese version of Bedtime Procrastination Scale.
